# Similar activation of sputum granulocytes in eosinophilic and non-eosinophilic asthma

**DOI:** 10.1186/2045-7022-5-S2-O8

**Published:** 2015-03-23

**Authors:** Bart Hilvering, Tamar Tak, Kiki Tesselaar, Leo Koenderman

**Affiliations:** 1 University Medical Centre Utrecht, Respiratory Medicine, Lab. of Trans. Immunology, Utrecht, Netherlands; 2 University Medical Centre Utrecht, Immunology, Lab. of Translational Immunology, Utrecht, Netherlands

## Background

Inflammatory phenotypes of asthma are associated with differences in disease characteristics. It is however unknown whether clinical differences between patients with eosinophilic and non-eosinophilic asthma are reflected by differential cell surface expression patterns of neutrophils and eosinophils in blood compared to sputum.

## Method

We obtained peripheral blood and induced sputum from 21 asthma patients and defined two patient groups based on sputum eosinophilia (cut-off 3%). Eosinophils and neutrophils from blood and sputum were analysed by flow cytometry for expression of activation and degranulation markers. Data were analysed by both classical, non-parametric statistics and a more advanced multi-dimensional approach, using principal component analysis (PCA).

## Results

Patients with eosinophilic asthma (>3% sputum eosinophils) were characterised by increased ACQ scores and blood eosinophil counts. Both sputum neutrophils and eosinophils displayed a highly activated and degranulated phenotype compared to cells obtained from blood. More specifically, degranulation of all granule types was detected in sputum neutrophils and eosinophils, combined with an increased expression of the activation markers (activated) Mac-1, Programmed Death-Ligand 1 (PD-L1) and a decreased expression of L-selectin. CD69 expression was only increased on sputum eosinophils. Surface marker expressions did not differ between patients with eosinophilic and non-eosinophilic asthma, neither by single nor by multi-dimensional analysis.

## Conclusion

The main finding is that granulocytes are highly activated and degranulated in sputum and that expression profiles of activation and degranulation markers on sputum granulocytes were similar for patients with eosinophilic and non-eosinophilic asthma. Therefore, we conclude that clinical differences between eosinophilic and non-eosinophilic asthma patients are most likely not reflected by differences in granulocyte activation in sputum. Secondly, we are the first to report the expression of an immune-suppressive receptor PD-L1 on sputum granulocytes, suggesting an immuno-modulatory role of these cells in inflamed airways.

